# Inhibition of B cell receptor signaling induced by the human adenovirus species D E3/49K protein

**DOI:** 10.3389/fimmu.2024.1432226

**Published:** 2024-07-30

**Authors:** Andreas Hildenbrand, Precious Cramer, Milena Bertolotti, Nathalie Sophia Kaiser, Kathrin Kläsener, Clara Muriel Nickel, Michael Reth, Albert Heim, Hartmut Hengel, Hans-Gerhard Burgert, Zsolt Ruzsics

**Affiliations:** ^1^ Institute of Virology, Medical Center and Faculty of Medicine, University of Freiburg, Freiburg, Germany; ^2^ Spemann Graduate School of Biology and Medicine (SGBM), University of Freiburg, Freiburg, Germany; ^3^ Signaling Research Centers CIBSS and BIOSS, University of Freiburg, Freiburg, Germany; ^4^ Navita S.r.l., University of Eastern Piedmont A. Avogadro, Novara, Italy; ^5^ Department of Rheumatology and Clinical Immunology, Medical Center University of Freiburg, Faculty of Medicine, University of Freiburg, Freiburg, Germany; ^6^ Institute of Virology, Hannover Medical School, Hannover, Germany

**Keywords:** adenovirus, E3/49K, immunoevasion, BCR signaling, CD45

## Abstract

**Introduction:**

The early transcription unit 3 (E3) of human adenoviruses (HAdVs) encodes several immunoevasins, including the E3/49K protein, which is unique for species D of HAdVs. It is expressed as surface transmembrane protein and shed. E3/49K of HAdV-D64 binds to the protein tyrosine phosphatase surface receptor CD45, thereby modulating activation of T and NK cells.

**Methods:**

Considering that E3/49K represents the most polymorphic viral protein among species D HAdVs, we demonstrate here that all tested E3/49K orthologs bind to the immunologically important regulator CD45. Thus, this feature is conserved regardless of the pathological associations of the respective HAdV types.

**Results:**

It appeared that modulation of CD45 is a unique property restricted to HAdVs of species D. Moreover, E3/49K treatment inhibited B cell receptor (BCR) signaling and impaired BCR signal phenotypes. The latter were highly comparable to B cells having defects in the expression of CD45, suggesting E3/49K as a potential tool to investigate CD45 specific functions.

**Conclusion:**

We identified B cells as new direct target of E3/49K-mediated immune modulation, representing a novel viral immunosubversive mechanism.

## 1 Introduction

Mucosal infections with human adenoviruses (HAdVs) are highly prevalent (1). In human populations worldwide and at all ages HAdV infections represent a significant source for morbidity causing a broad range of severity, including rare fatal cases especially in infants and immunocompromised individuals (2). Phylogenetically, HAdVs belong to the genus *Mastadenoviridae* and are classified into seven species (A-G) comprising more than 100 known types (3–6). Each HAdV species causes distinct profiles of clinical symptoms related in part to specific tissue tropisms and entry receptor utilization (7–9). By far the largest species comprises the species D (5), which infects exclusively humans (10) and is reported to be responsible for a variety of ocular pathologies (9).

While the genomes of HAdVs are well conserved among the different species, most pronounced among the structural proteins, all HAdVs contain a relatively variable region, called early transcription unit 3 (E3). While this region is rather conserved within each species (11), it differs significantly between species encoding a number of different gene products (12, 13). Interestingly, the E3 region is not required for viral replication *in vitro* and therefore can be deleted without affecting virus replication in tissue culture (14, 15). However, *in vivo* the E3 is suggested to play an important role in viral fitness and pathogenesis, as E3 is present in all HAdVs and encodes gene products with immunomodulatory functions (12, 16–18) that seem to affect xenograft transplant survival (19) and clinical effectiveness of recombinant oncolytic HAdVs (20).

The largest group of E3 proteins belongs to the protein family of conserved region 1 (CR1) viral proteins, consisting mainly of type I membrane glycoproteins. Multiple members of this family can be found in human and primate AdVs as well as in human and primate cytomegaloviruses (here coined *RL11* family). It can be assumed that these genes originate from a common ancestor and have evolved separately to execute diverse functions for the distinct viruses (21).

The individual sets of species-specific CR1 open reading frames (ORFs) show unique nucleotide compositions whereas some restricted interspecies homologies are observable. Due to this relation the diverse species can be grouped based on their E3 sequence composition and association with some group- specific virus-host interactions and diseases patterns (12, 13, 17, 21). To date, the functional knowledge about the CR1 proteins is still limited. The species D CR1-β protein, also named E3/49K, for its predicted molecular weight of 49kDa (12), is unique for this species. It was among the first family members whose functional activity could be characterized. E3/49K is the largest member of the adenoviral CR1 family with an apparent molecular weight on SDS PAGE of 80–100 kDa due to its extensive glycosylation (21). This protein has been extensively studied for HAdV-D64, an EKC causing type of species D HAdVs, which was previously called Ad19a. Like most of the other CR1 proteins E3/49K is a type I transmembrane protein starting to be expressed at early stages of infection, but continues to be expressed throughout the infection cycle (12, 13, 21, 22). At steady state, the E3/49K protein of HAdV-D64 is shuttling between the cellular compartments of the endoplasmic reticulum, trans-Golgi network, plasma membrane, and endosomes, eventually accumulating at late stages of the infection in the lysosome (22, 23). At the cell surface the ectodomain (ECD) of E3/49K is proteolytically processed close to the transmembrane region by matrix metalloproteases, possibly involving both ADAM-10 and ADAM-17, resulting in the shedding of a 60–80 kDa extracellular fragment of the CR1-β protein. Shed E3/49K (sec49K) represents the only secretory HAdV protein known to date (23, 24). It is reported, like the membrane anchored version, to bind to its host receptor, the surface protein tyrosine phosphatase CD45 (23–25). Encounter with soluble E3/49K inhibits the activation of human T and NK cells (23, 24, 26, 27).

During lymphocyte activation, the surface ligation by cognate antigens initiates the intracellular signal transduction, involving the orchestration of different downstream signaling pathways (28–30). CD45 is a critical regulator in lymphocyte receptor signaling and primes lymphocytes by removing inhibitory phosphate groups on src-family kinases (SFKs), which were mainly analyzed in T cells of human blood samples and in cell lines as well as mouse models (27, 31–33). Interestingly, neither the regulatory mechanism behind the control of CD45 activity nor any physiological ligand for the extracellular ECDs of CD45 has been established to date. What complicates matters further is the expression of distinct alternatively spliced CD45 isoforms on all leukocyte types. However, its fundamental role within the immunological network has been clearly established. This notion is supported by T and B cell dysfunctions resulting in life-threating severe combined deficiency syndromes (SCID) in patients lacking CD45 (34–37). Consistent but also opposing findings were made in permanent genetic mouse models with various gene deletions (27, 38, 39). Thus, one can raise the question whether findings from mouse CD45 models can be automatically applied to humans. This underlies the need for a tool to mimic CD45 deficiency in humans allowing the detailed analysis of CD45 in primary human cells (27).

This is especially evident for B cells, in which the role of CD45 has been poorly defined and appears to be more complex. In CD45 deficiency, T lymphocytes are more profoundly affected compared to B lymphocytes resulting in an almost complete absence of T cells in patients and mice lacking CD45. The reason seems to be the severe impairment of T cell receptor (TCR) signaling resulting in a drastic loss of T cells during thymic development. By contrast, peripheral B cell numbers are actually elevated in CD45 deficiency (39–43). While B cells from CD45 knockout mice also exhibit abnormal BCR functions, but the impact is less severe relative to T cells (43–45). In the mouse model, serum immunoglobulin levels were relatively normal (46), but decreased with age in a reported SCID child (35). In addition, B cells from CD45-deficient mice respond normally to T cell-dependent and -independent stimuli (39, 41, 43–45). These reports indicate that CD45 may have different roles in the development and functions of T and B cells.

Characterizing the function of E3/49K of HAdV-D64 in T cells revealed that the abolished T cell functions resulted from the inhibition in the lymphocyte receptor signaling machinery (24, 25). Since E3/49K was shown to bind to (24) and modulate B cell activation (47), it was suggested that these are direct effects of E3/49K on B cells rather than a consequence of its known effect on T cells (24, 25). Therefore, the direct impact of E3/49K on human B cells was further investigated in this study. Remarkably, the ECD of the species D CR1-β protein exhibits the highest polymorphism of the entire proteome of species D HAdVs, suggesting this protein to be under enormous evolutionary pressure to vary, what is likely for a soluble viral protein due to the high exposure to immunological interference mechanisms (11). A recent study by Martinez-Martin suggested that Fc-tagged ECDs of the species D CR1-β proteins may bind apart from CD45 to other host cell surface proteins. Furthermore, Fc-tagged E3/49K proteins from additional species D types appeared to bind to CD45 too (48).

The presented study confirms the conserved activity of differing species D CR1-β products. Due to its immunomodulatory functions it was previously speculated that E3/49K of HAdV-D64 might be involved in the disease process of the severe eye disease, epidemic keratoconjunctivitis (EKC) (24, 48). To investigate this further, several virus types from species D adenoviruses were selected that were either associated with EKC or not to enable a comparative analysis at the functional level. To avoid the use of different reagents for the different HAdV-D types, we expressed N-terminally HA-tagged and codon-optimized E3/49K (HA-49K) orthologs in stably transfected A549 cells. Subsequently, the effect of the different orthologs was compared in various cell-based assays. In addition, usage of soluble CD45, allowed to assess the presence of a potential CD45-binding activity in species other than species D during HAdV infection. Moreover, we identified that targeting of B cells with E3/49K inhibits the BCR signaling. We demonstrated that E3/49K mediates inhibition of MAPK pathway signaling which is in agreement with previous observations in CD45-deficient mice (43, 45, 49). On this basis we suggest E3/49K as potential tool to investigate CD45-specific functions in human cells by inhibiting CD45 activity, which can be controlled by addition of decoy receptors.

## 2 Materials and methods

### 2.1 Cloning and molecular biology methods

Synthetic HA-tagged and codon-optimized E3 CR1-β protein coding sequences of HAdV-D8 (Freiburg strain, GenBank Accession Nr.: KP016737), -D19 (strain AV-587, AB448771), -D36 (strain USA, GQ384080.1), and -D64 (ME strain, CS301726) were cloned into the multiple cloning site of the pSG5 expression vector as before (22), using here EcoRI and BamHI sites resulting in pSG5-D8-HA-49K, pSG5-D19-HA-49K, pSG5-D36-HA-49K, and pSG5-D64-HA-49K, respectively. For codon optimization of E3/49K constructs the service of GeneArt (Thermo Fisher Scientific) was utilized. The HA-tag was inserted into the optimized coding sequences N-terminally to the first amino acid of the predicted mature protein after signal sequence cleavage (Gly20 for HAdV-D8, -D19, -D64, and Asp21 for -D36). The expression plasmids pSG5-D64–49KCO and pSG5-D64–49KCO-AAA encoding the untagged control proteins, the wild-type E3/49K and E3/49K-YA/LLAA mutant, respectively, were constructed in the same way, but the codon-optimized E3/49K coding sequence of HAdV-D64 was inserted into the pSG5 vector either unchanged or with a mutated tyrosine-based YxxΦ sorting motif encoding alanine instead of Tyr416, Leu423, and Leu424 codons (23).

### 2.2 Cell culture, viruses and infection

A549 (ATCC, CCL-185) and 293A cells (Invitrogen R70507) were maintained in Dulbecco’s modified Eagle’s medium (DMEM) (Gibco (Life Technologies)) supplemented with 10% (v/v) fetal calf serum (FCS) (PAN-Biotech GmbH), 100 U/ml penicillin (Gibco (Life Technologies)), 0.1 mg/ml streptomycin (Gibco (Life Technologies)) and 2 mM L-glutamine (Sigma-Aldrich). We also established as control A549-based cell lines expressing codon-optimized but untagged E3/49K (A549E3/49K CO7) and the tail-mutant YA/LLAA (23) of HAdV-D64 described earlier. For cultivation of stably transfected A549 cells additional 0.5 mg/ml G418-disulphate (Formedium) was added to the medium for A549 cells. The lymphoid cell lines Jurkat E6–1 (ATCC, TIB.152TM) and its CD45 knockout variant J45.01 (32), Ramos (ATCC, CRL-1923) and Ramos CD45 knockout cells were cultivated in RPMI1640 medium ((Gibco (Life Technologies)) supplemented with 10% FCS, 100 U/ml penicillin, 0.1 mg/ml streptomycin, and 2 mM L-glutamine. The viruses HAdV-A12, -B3, -B35, -C5, -D8, -D19, -D36 were obtained from the German Adenovirus Reference Laboratory, Hannover Medical School, Germany. HAdV-D64 (50), -D64ΔE3 (50), -D64ΔE3 + 49K (50) have been constructed as described previously and their properties published (23, 50). HAdV-E4 was obtained from ATCC. All HAdVs were propagated in either A549 or 293A cells. Viral titers were assessed via the AAV-Gluc-B3 based conditional reporter expression system (51). Routinely, A549 cells were infected with an MOI of 5 for 24 h. A productive infection was performed, orientated on flow cytometry analysis on the basis of an efficient detection of hexon capsomers.

### 2.3 Generation of the Ramos CD45 knock out cell-line

Ramos WT cells with engineered B cell receptor (52) were used to generate Ramos CD45 knockout cell line with gRNA CD45.1: ACAACCACTCTGAGCCCTTC – TGG (target exon 7) and gRNA CD45.2: GTATTTGTGGCTTAAACTCT – TGG (target exon 2). CRISPR/Cas9 deletion of CD45 was carried out using the Neon Transfection System (Invitrogen) to deliver the ribonucleoprotein (RNP) into the cells according to the genome editing method of Integrated DNA Technology (IDT). In brief: Ramos cell medium was changed the day before transfection. 1x 10^6^ Ramos cells were pelleted by centrifugation and resuspended in 9 μL buffer R (Neon), 1 µL of RNP complex, and 2 µL of electroporation enhancer (IDT). RNP complex contained diluted sgRNA (annealed crRNA and tRNA in a 1:1 ratio in IDTE buffer) and Cas9 endonuclease in buffer R (NEON). Electroporation was performed in 10 µL NEON tips at 1350 V, 30 ms, with a single pulse. The transfected cells were first recovered for 72 h at 37°C and 5% CO_2_ without antibiotics and then subjected to complete RPMI medium. The Ramos CD45 knockout cell line was controlled in parallel by transfection of a negligible protein (p62). Successful transfected Ramos cells were batch sorted using BioRad cell sorter in three different rounds. Deletion of the target gene was verified by flow cytometry.

### 2.4 Stable transfection of A549 cells and production and purification of soluble E3/49K

For generation of stable HA-49K cell lines expressing the proteins derived from HAdV-D8, -D19, -D36 and -D64 A549 cells were stably transfected via electroporation with linearized pSG5 vectors containing HA-49K constructs together with the linearized plasmid of pGCneo-635 containing the resistance gene for G418-disulphate. Electroporation was performed with the Gene Pulser Xcell Electroporator (Bio-Rad Laboratories, Inc.) at 227 V, 960 µF and ∞ Ω. Selection of G418-resistant cell clones was executed by cultivation in cell culture medium containing 1 mg/ml G418-disulphate and utilization of cloning cylinders. To create HA-49K containing supernatants stable cell lines were cultivated in 15 cm Ø dishes until reaching full confluence. Plates were washed once with PBS and then further incubated for 10 days with FCS-free DMEM. Cell supernatants were collected by centrifugation at 2,000 g for 10 min at 4°C to get rid of cell debris. Subsequently, supernatants were stored at 4°C. For HA-49K protein purification, cell supernatants were incubated for 1 h rolling at 4°C with Pierce^®^ α -HA agarose (Thermo Fisher Scientific) and eluted using 3 M sodium thiocyanate. Purified proteins were obtained after rebuffering in PBS utilizing centrifugal concentrators (Vivaspin 20, 30.000 MWCO PES, Sartorius AG), and filtration with 0.2 µm syringes filters (Pall Corporation). Thereafter, aliquots were frozen at -80°C. A selection of the best producer cell clone for each HA-49K type was performed using flow cytometry of cell surface expressed HA-49K and target cell binding capacity as described below.

### 2.5 Cell lysis, SDS-PAGE, western blot analysis

Cellular protein extracts were generated by lysis of cells using 50–100 µl of cell lysis buffer (5 mM MgCl_2_, 20 mM Tris-HCl, 140 mM NaCl, 1% NP-40, cOmplete™ Protease Inhibitor Cocktail (Roche AG)). The cell suspension was frozen for 24 h at -80°C. After thawing, the suspension was centrifuged at full-speed for 20 min at 4°C for separation of the extract. Proteinaceous solutions were further processed with SDS-PAGE and subsequently analyzed via immunoblotting. For immunoblotting proteins were transferred from SDS-PAGE to nitrocellulose membrane via western blotting followed by immune detection. Detection of HA-tagged proteins was performed, using an α-HA Ab. To control detection of HA-49K, the HA-tagged multi-tag (MT) control protein was used (Absolute Antibody). Blot development was performed with utilization of SignalFire™ ECL Reagent (Cell Signaling Technology) and monitored using Odyssey^®^ FC Imaging System (LI-COR Biosciences GmbH).

### 2.6 Flow cytometry, E3/49K binding assay, hCD45-Fc binding assay, calcium flux assay and competition assay

Flow cytometry measurements were performed with 400,000 infected or non-infected A549 cells, stable transfectants or E3/49K target cells. E3/49K binding assays were executed as described previously (23–25). For detection of bound E3/49K versions an indirect antibody (Ab) staining procedure was conducted. Abs were incubated for 45 min at 4°C. HA-49Ks were detected via α-HA or 4D1 (24) monoclonal (mAbs), whereas for untagged E3/49K only the 4D1 Ab was used. To control detection of HA-49K, the HA-tagged MT control protein was used (Absolute Antibody). Cell fixation using 4% paraformaldehyde was performed for HAdV infected cells as well as for the assessment of cell surface upregulated CD69 (24). Following fixation cells were quenched with utilization of 50 mM NH_4_Cl. For internal staining, the buffer (PBS, 3% FCS) was supplemented with 0.1% saponin. To quantify the binding activity to human CD45, infected cells or HA-49K producer cell lines were treated with 0.5 µg/sample of recombinant human CD45-ECD with an IgG1 Fc-tag (hCD45-Fc) (Sino Biological). Afterwards, CD45 binding was detected via α-panCD45 mAbs MEM-28 and GAP8.3 (24). Binding activity of the HA-49Ks was competed with an untagged E3/49K of HAdV-D64 in consecutive incubations. To prevent E3/49K functions, it was previously incubated for 30 min with hCD45-Fc decoy receptors.

For assessment of the calcium flux response, 1x10^6^ Ramos cells, pre-incubated with HA-49Ks, were resuspended in 1 ml full medium supplemented with 15 μl Indo-1 AM (Invitrogen) and incubated for 45 min at 37°C. Subsequently, Ramos cells were washed two times with PBS and resuspended in 1 ml full medium directly before the calcium flux measurement. First, basal calcium levels were acquired for 1 min. Subsequently, 10 µg/ml α-human IgM was added to the cells for stimulation and calcium levels were assessed for additional 3 to 4 min. Flow cytometry measurements were executed with BD FACSCanto™ II or BD LSRFortessa™ instruments from BD Bioscience. The data were evaluated with the FlowJo^®^ v10.07.1 software from BD Bioscience.

### 2.7 Jurkat and Ramos cell stimulation

For phosphorylated extracellular signal-regulated kinases 1 and 2 (pErk1/2) analysis, Jurkat cells were stimulated with 1 µg/ml α-CD3 mAbs and Ramos cells with 1 µg/ml α-human lambda-L F(ab) fragment (SouthernBiotech). Stimulation was performed with 2.5x 10^5^ cell per sample for 2 min at 37°C and stopped by washing with cold PBS. pErk1/2 detection was performed by immunoblotting analysis using α-human pErk1/2 mAbs. To measure CD69 upregulation 4x10^5^ Jurkat cells per sample were stimulated via 5 µg/ml immobilized α-CD3 and soluble 1 µg/ml α-human CD28 mAbs for 6 h at 37°C. Stimulation was terminated by washing with cold PBS. Subsequently, cells were fixed as described above.

### 2.8 Primary B cell purification, stimulation and lysis

Human peripheral blood mononuclear cells (PBMCs) were isolated from whole blood derived buffy coats, which were isolated by density gradient centrifugation using lymphocyte separation medium (density: 1,077 g/ml, Anprotec) according to manufacturer’s instructions. The buffy coats were taken off carefully and washed with 2 mM EDTA in PBS solution. Then, the B cells were isolated with the EasySep Human B cell isolation kit (STEMCELL Technologies) according to the manufacturer’s protocol. For stimulation of primary B cells, IgG positive B cells were stimulated with 10 µg/ml α-human IgG (Jackson Immuno Research) for 5 or 10 min at 37°C. After that, stimulation was terminated immediately by washing with cold 2 mM EDTA in PBS solution and centrifuged at 400 g for 7 min at 4°C. Cell were later lysed in RIPA Lysis and Extraction Buffer (Thermo Fisher Scientific) and the lysate stored at -20°C for downstream experiments.

### 2.9 Antibodies

For flow cytometry staining of E3/49K 4D1 mAb or α-HA (HA-7, Sigma-Aldrich) Abs were used. For stimulation of Jurkat cells α-human CD3 (OKT3, BioLegend) and α-human CD28 mAbs were used (BD Biosciences). Isotype control IgG1 and isotype control IgG2 Abs were used from BD Biosciences. α-human CD69 mAbs (Miltenyi Biotec) were utilized to assess activation levels. For staining of human CD45, two pan-CD45 mAbs, MEM-28 (Biomol) and GAP8.3 (53) (kindly provided by Dr. Peter Cresswell, Yale University), were applied. Ramos cells were stimulated via α-human lambda-L F(ab’)2 from SouthernBiotech. Infection efficiency was quantified by staining for HAdV hexon capsid components using mAb 2Hx-2 (hybridoma supernatant, ATCC HB-8117). Human IgG positive B cells were stimulated via α-human IgG (Jackson Immuno Research) polyclonal Abs (pAbs) and identification of the B cell population was achieved with a α-human CD19 (HIB19) mAbs from BioLedgend. In the indirect staining setting rat Abs were detected with α-rat IgG (Thermo Fisher Scientific) and mouse derived Abs recorded with pAb α-mouse IgG (BD Biosciences) or mAbs (Thermo Fisher Scientific). For immunoblotting analysis β-actin was used as loading controls as detected with α-β-actin mAb (AC-74) from AC-74 Sigma-Aldrich, α-human IgG (Jackson Immuno Research) and α-human spleen tyrosine kinase (Syk) mAbs (C87C1, Cell Signaling Technology). Phosphorylated human Syk (pSyk) was detected with a mAb from BD Biosciences and human pErk1/2 with mAb from Santa Cruz Biotechnology.

### 2.10 Statistics

For statistical analysis the two-way ANOVA test was applied. Analysis and graphical illustrations were performed via GraphPad PRISM^®^ Version 8.1.0 (GraphPad Software Inc.). Flow cytometric measurement was evaluated with FlowJo^®^ v10.07.1 (FlowJo, LLC).

## 3 Results

### 3.1 The A549-based expression system provides processed products of HA-49K orthologs

**Figure 1:**
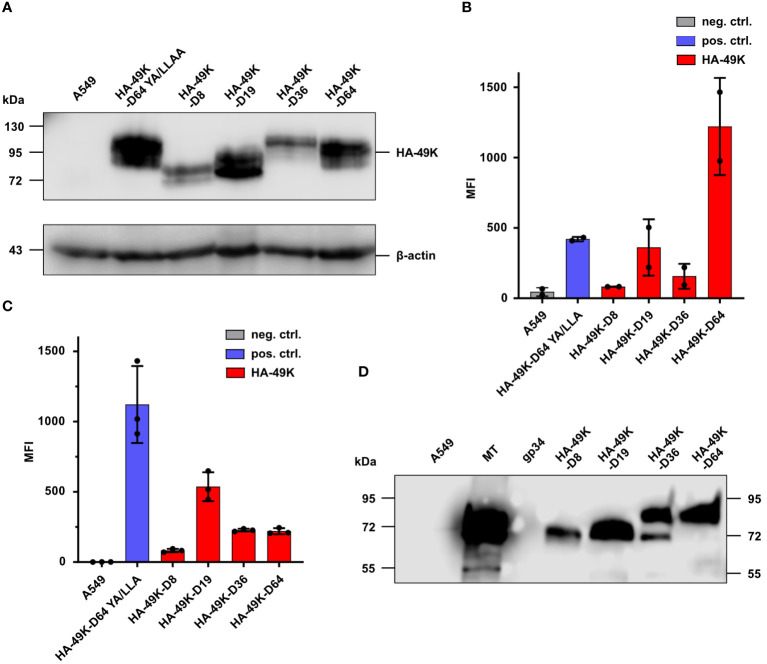
Stable A549 transfectants express HA-49Ks both in intracellular and extracellular compartments. Selection of a set of producer cell lines for HA-49K of HAdV-D8, -D19, -D36, and -D64 was previously carried out by quantitative flow cytometric analysis in at least three independent experiments. Untransfected A549 cells and the stable transfectant expressing a tail-mutant version of HA-49K of HAdV-D64 YA/LLAA (23) were used as negative and positive controls. 1 µg MT and recombinant His-tagged gp34 from HCMV (54) were utilized as protein controls in HA-based analysis. Cell lines were lysed and HA-49K expression examined via SDS-PAGE and immunoblotting using α-HA Abs **(A)**. Intracellular **(B)** and cell surface **(C)** expression levels of HA-49K were determined by flow cytometry. The columns represent the mean fluorescence intensity (MFI) obtained in independent experiments, each depicted as dots, error-bars represent the standard deviations. Equal volumes of cell supernatants from the cell lines indicated were collected after 10 days of cultivation and examined for soluble HA-49K using HA-specific Abs and SDS-PAGE followed by immunoblotting **(D)**.

To date, the biochemical characteristics and the functional activity of E3/49K is only described for HAdV-D64. Regarding the other types of HAdV-Ds or other species, there are no data existing. Windheim et al. (24) demonstrated that E3/49K is able to bind to any CD45-expressing cell type. But today, the mechanism of E3/49K caused immunomodulation, is merely described for T- and NK cells. Species D CR1-β gene products are unique for this species and differ significantly between different types. Interestingly, species D shows also a type-specific disease spectrum, which may be related to the distinct biochemical and/or functional properties of individual E3/49Ks. To examine any putative relationship between these phenomena and EKC disease four divergent E3/49K orthologs were selected from HAdV-D8 and -D64 causing EKC and -D19 and D36 not-causing EKC. Constructs of HA-49K orthologs were prepared via cloning as described in the method section and protein products were generated via stably transfected A549 cells. HA-49K expression by producer cell clones was probed by Western blot analysis (**Figure 1A**). The band positions and apparent MWs correlated with the number of predicted N-glycans, meaning that HA-49K of HAdV-D36 and -D64 with predicted 14 N-glycans, showed the highest molecular weight, compared to -D19 and -D8 proteins with 11 and 10 predicted N-glycans, respectively. The presence of HA/49K orthologs was quantitatively assessed also via intracellular flow cytometry measurement in comparison to the tail-mutant HA-49K –D64 YA/LLAA (23) (**Figure 1B**). As previously described for E3/49K of HAdV-D64 (22), all different orthologs are expressed on the cell surface of transfected clones (**Figure 1C**), although at different levels. This may be especially important for the shedding process as described before for E3/49K of HAdV-D64. For this reason, supernatants of HA-49K producer cells were collected and examined for the presence of soluble HA-49K versions by western blot analysis. Release of HA-49K was indeed demonstrated case for all types (**Figure 1D**), indicating that shedding of the ECD is a common property to all HA-49Ks tested. Overall, the A549 expression system seems to provide sufficient protein products of any HA-49K ortholog.

### 3.2 E3/49K orthologs share conserved binding activity for CD45 receptors

**Figure 2:**
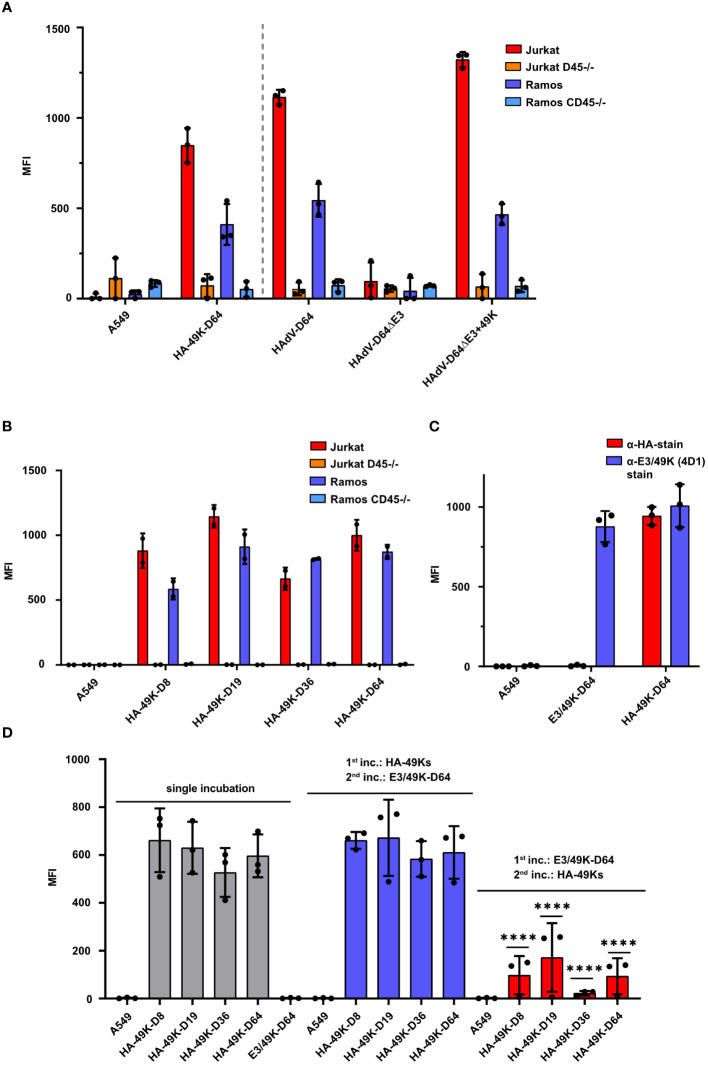
HA-49K orthologs from the A549-based expression system function comparably to natural E3/49K and exhibit a consistent binding activity to CD45 expressing target cells. Cell supernatants containing individual E3/49K variants were incubated together with target cells and bound E3/49K was measured via flow cytometry. Binding activity of HA-49K of HAdV-D64 from the A549-based producer cell line was compared to E3/49K obtained from cells infected with HAdV-D64, HAdV-D64ΔE3 and HAdV-D64ΔE3 + 49K viruses (49). Supernatants were incubated with wild-type Jurkat (red), Ramos (blue) and the CD45-deficient Jurkat (orange) and Ramos (light blue) cells, respectively. The grey dashed vertical line separates results obtained from transfected cells (left) from those of infected cell lines (right). Binding of HA-49K and E3/49K was detected with 4D1 mAb **(A)**. Binding activity to Jurkat and Ramos cell lines of recombinant HAdV-D64 HA-49K of was compared with HA-tagged orthologs of HAdV-D8, -D19 and -D36, respectively. Binding was determined with the target cell system as applied in the previous experiment using α-HA Ab **(B)**. Contrasting the binding of HA-49K and untagged E3/49K from the A549-based expression system to Jurkat cells reveals no negative influence to the binding activity by the HA-tag. Bound E3/49K versions were detected using HA-specific (blue) or E3/49K-specific (4D1, red) mAbs **(C)**. The binding specificity of HA-49K orthologs was further characterized by competition with untagged E3/49K of HAdV-D64. Residual binding activity of HA-49K orthologs was monitored by flow cytometry using α-HA Abs and a two-step sequential incubation of Jurkat cells with supernatants, containing E3/49K variants. The order of the individual incubations for competition is indicated within the figure. Significant differences to single incubations were analyzed **(D)**. Cell supernatants from normal A549 cells were utilized as negative controls. The columns represent the mean-MFIs obtained in independent experiments, each depicted as dots, and error-bars represent the standard deviations. Statistical significance (****P<0.0001) was determined via the two-way ANOVA test and is indicated within the panel **(D)**.

Previous publications demonstrated that recombinant E3/49K of HAdV-D64 produced in the A549 expression system is shed into the cell supernatant and binds to CD45 expressing target cells (24). According to metabolic labeling experiments with infected cells, shed E3/49K was also found in the cell supernatant as well (22), suggesting that both the viral and the recombinant sec49K bind to CD45 expressing cells *in trans*. To verify that the maintained activity of recombinant HA-49K is similar to that of nascent E3/49K produced during viral infection, we collected cell supernatants from the A549-based expression systems and from infected cells and incubated them with Jurkat T cell and Ramos B cell lines, which either express CD45 or do not (CD45-/-). Supernatants from cells infected with E3/49K competent HAdV-D64 or HAdV-D64ΔE3 + 49K viruses (50) as well as from the HA-49K producer cell line exhibited a comparable binding activity to both target cells. Overall, the binding activity to Jurkat cells tended to be stronger compared to the binding to Ramos cells, correlating with the CD45 surface expression levels of these cell lines (**Supplementary Figure 1A**). No E3/49K binding at all was detected for both CD45-deficient cell lines which were comparable to the controls of A549 cells or upon infection with HAdV-D64ΔE3 which does not express E3/49K (50) (**Figure 2A**). Thus, the A549-based expression system provides functional products comparable to natural proteins produced during infection. Including HA-49K of the types -D8, -D19 and -D36 into the target cell binding system reveals a preserved specific functional activity. Depending on the expression of CD45 all tested proteins were bound to T and B cell lines to a similar extend (**Figure 2B**). Thus, the HA-tag is not affecting the binding activity to CD45, since untagged and tagged recombinant E3/49K of HAdV-D64 showed comparable binding levels (**Figure 2C**). Such untagged E3/49K was used for further characterization of the binding activity of the different orthologs. By competing the untagged E3/49K of the type -D64 with the HA-tagged version in a two-step sequential incubation series, applying an initial incubation with untagged E3/49K and a subsequent incubation with HA-49K, resulted in a drastically reduced binding activity of HA-49K in the Jurkat target cell binding system. Previous incubation of untagged E3/49K of HAdV-D64 is also blocking significantly the binding activity of the other HA-tagged orthologs, revealing a conserved binding site of HA-49Ks on CD45 receptors (**Figure 2D**). Since the replacement levels are correlating with the overall HA-49K content of the individual supernatant (**Supplementary Figure 2**), it can be assumed that the tested orthologs share similar binding affinities.

### 3.3 Inhibition of leukocyte activation by HA-49K orthologs is conserved functional

**Figure 3:**
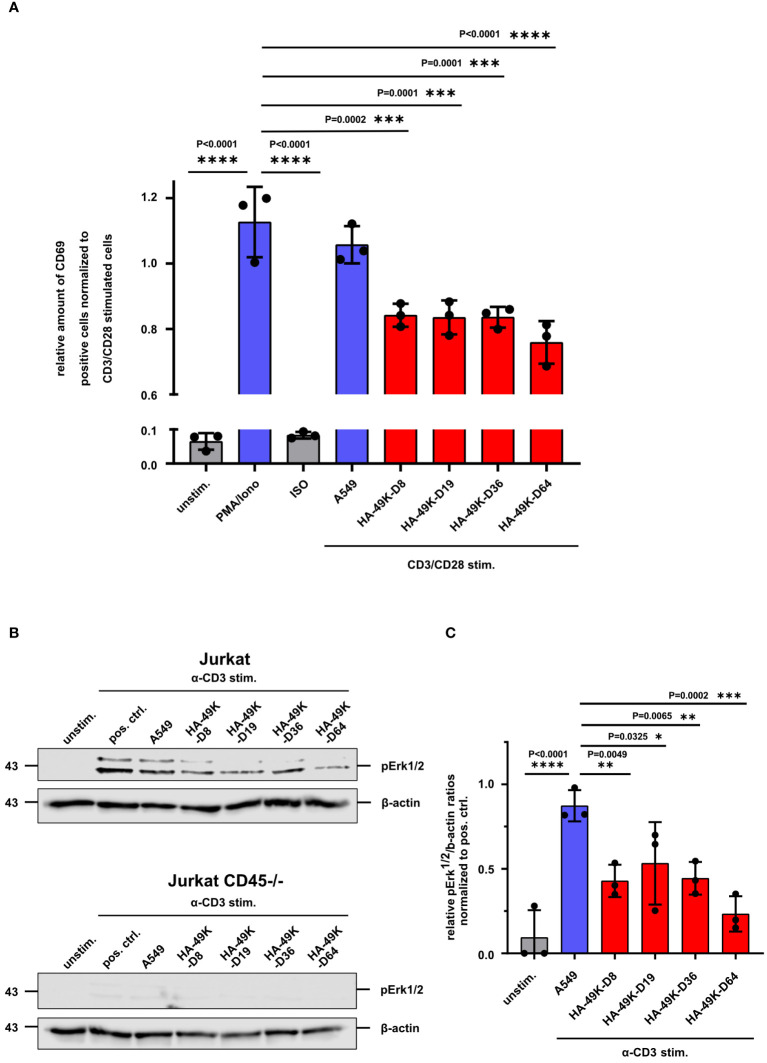
Activation of Jurkat T cells is inhibited by HA-49K orthologs to an equal extent. Jurkat cells were previously incubated with cell supernatants (red) containing HA-49K orthologs. Cells were washed and stimulation was conducted via receptor cross-linking using immobilized α-CD3 and soluble α-CD28 Abs for 6h. After cell fixation the activation level was determined by flow cytometry-based monitoring of the cell surface expression of the early activation marker CD69. Relative numbers of CD69 positive cells were normalized to CD3/CD28 stimulation control (not shown). Untreated (unstim.) and isotype control (ISO) treated samples were used as negative controls (grey). To show the efficiency of the CD3/CD28 stimulation we treated the cells also with 50 ng/ml Phorbol-12-myristate-13-acetate and 1 µg/ml ionomycin (PMA/Iono, blue). Administration using CD3/CD28 stimulation and unreactive A549 supernatant was utilized to control the effect of the supernatant on CD3/CD28 stimulation (A549, blue) **(A)**. pErk1/2 levels were identified by immunoblot analysis upon CD3 stimulation of Jurkat cells with 1 µg/ml for 2 min. Sample loading was controlled by detection of β-actin. One representative blot for Jurkat and CD45-/- Jurkat is shown **(B)** and the relative expression levels of pErk1/2 to β-actin ratios were normalized to the pos. ctrl. **(C)**. The columns represent the mean of 3 individual experiments (dots), the error-bars represents the standard deviation for A and **(C)** Statistical differences compared toPMA/ionomycin treatment in **(A)** and CD3-stimulation in the presence of A549 supernatants in **(C)** positive controls were analyzed using the two-way ANOVA test. Only significant results were indicated in the figure.

Prior studies showed that Jurkat cells treated with E3/49K express lower levels of the early activation marker CD69 and pErk1/2 upon CD3 cross-linking (24, 25). Having determined a conserved binding property for all tested HA-49K types, we asked whether these potential immunoevasins also exhibit a similar activity to modulate immune cells. As before for E3/49K of HAdV-D64, we tested the functions of recombinant HA-49Ks first in the Jurkat T cell model. After pre-treatment of Jurkat cells with HA-49K containing supernatants and CD3/CD28 stimulation, the number of activation marker CD69 positive cells were quantified using flow cytometry. Similar to E3/49K of HAdV-D64 also the other tested HA-49K types caused a reduction of CD69 levels to a comparable magnitude by some 30% (**Figure 3A**). To verify this immune suppressive effect of HA-49Ks, we tested their role in the modulation of TCR signaling after binding to CD45. CD45 deficiency causes an overall impairment of TCR transduction in Jurkat cells, preventing e.g. downstream signals dependent on the mitogen-activated protein kinase (MAPK) pathway (**Figure 3B**) (32, 55, 56). Accordingly, treatment of Jurkat cells with HA-49K containing supernatants resulted in a clear impairment of Erk1/2-phosphorylation upon activation via CD3 cross-linking to comparable levels across the tested types (**Figures 3B, C**). Thus, the published functional activity of HA-49K of the type -D64 could be reproduced and in addition also determined for the other tested types, suggesting a conserved biological activity for all species D CR1-β products.

### 3.4 BCR signaling of Ramos B cells is also targeted by E3/49K

**Figure 4:**
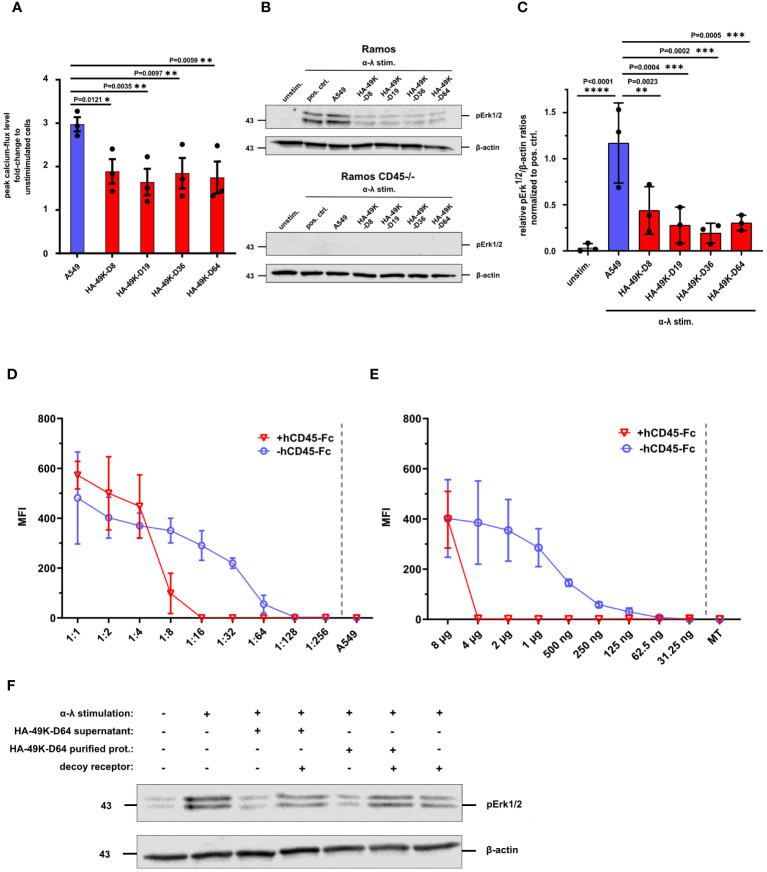
Ramos B cell signaling is inhibited by HA-49K orthologs to comparable levels. Ramos B cell signaling was determined to assess the inhibitory potential of HA-49K orthologs in B cells. Previous incubation of Ramos cells with cell supernatants (red) containing various HA-49K orthologs was performed. Unstimulated cells (unstim.) were used as a negative control (grey), co-incubation with unreactive A549 supernatant (A549) served as a positive control indicated in blue. Cells were washed and stimulated via receptor cross-linking using α-λ Abs. The cellular calcium-response was detected by flow cytometry and the mean peak Ca2+-levels (columns) of 3 individual experiments (dots) including standard deviation are shown. Statistical differences compared to A549 were analyzed using two-way ANOVA test **(A)**. Immunoblot analysis of pErk1/2 levels upon BCR stimulation of Ramos B cell lines with 1 µg/ml α-λ Abs for 2 min. Unstimulated cells (unstim.) served as negative control while α-λ treated cells (pos. crtl.) and co-incubation with A549 supernatant (A549) served as positive controls. Detection of β-actin levels was used as loading control. One representative blot for Ramos and CD45-/- Ramos cells is shown **(B)**. The relative detection levels of pErk1/2 to β-actin ratios in Ramos cells were normalized to the positive ctrl. The mean (columns) of 3 individual experiments (dots) including standard deviation is shown. Statistical differences to the positive ctrl. were analyzed using the two-way ANOVA test. Only significant results were indicated in the panel **(C)**. A two-fold dilution series of cell supernatants containing HA-49K-D64 **(D)** and purified HA-49K-D64 proteins starting at 8 µg per sample **(E)** was performed. Samples were either supplemented with 0.5 µg per sample hCD45-Fc (+hCD45-Fc, red) or without (hCD45-Fc, blue). After a 1 h incubation period, the binding of HA-49Ks to Ramos cells was detected using α-HA-based flow cytometry. Undiluted supernatant from untransfected A549 cells (A549) or the 8 µg MT protein were utilized as negative controls and shown as single values at the end of the x-axis separated by the grey dashed line. The mean MFI of 3 individual experiments is displayed for each, including standard deviation in the form of error bars. Erk1/2 phosphorylation was analyzed to investigate the prevention of the inhibitory effect HA 49K-D64 by hCD45-Fc receptors via immunoblotting. The supernatant containing HA-49K-D64 proteins was diluted 1:10 and 4 µg purified HA-49K-D64 proteins were incubated for 1 h with 500 ng hCD45-Fc decoy receptors as indicated in the figure. Subsequently, reagents were incubated with Ramos cells for 1 h Cells were lysed after BCR stimulation with 2 µg/ml α-λ Abs for 2 min. Sample loading was controlled by the detection of β-actin levels. One representative blot is presented **(F)**.

E3/49K of HAdV-D64 inhibits T- and NK cell signaling and immune functions (24, 48). However, E3/49K of HAdV-D64 binds to all CD45 expressing leukocytes, including B cells (24). This scenario was confirmed here for the different HA-49K types tested (**Figure 2B**). Therefore, we asked whether B cells also serve as targets of E3/49K-mediated immune inhibition similar to T cells and focused here on BCR signaling. BCR signaling events were first investigated in Ramos B cells. BCR-mediated signaling is crucial for the activation and differentiation of B cells, involving several downstream signaling pathways (28, 29). Murine CD45-deficient B cells are characterized to exhibit defects in their calcium flux and MAPK pathway signaling (43, 45, 49). Therefore, Ramos cells were first pre-incubated with different HA-49K types and subsequently signaling events were tested after BCR cross-linking. Calcium flux measurements revealed reduced calcium flux responses compared to control after the stimulation of these HA-49K-treated cells (**Figure 4A**). Subsequently, we tested for Erk1/2 phosphorylation by Western blotting. Treatment of Ramos cells with different HA-49K types resulted in a clear impairment of pErk1/2 phosphorylation compared to controls (**Figures 4B, C**). Interestingly, in Ramos cells Erk1/2 phosphorylation seemed to be more sensitive to E3/49K mediated inhibition than in Jurkat cells. Comparable to CD45-deficient Jurkat cells (**Figure 3B**) Erk1/2 phosphorylation is absent in CD45-deficient Ramos cells (**Figure 4B**), suggesting that CD45 plays a crucial role in the regulation of the MAPK pathways in B cells. This E3/49K-mediated inhibition of MAPK pathway signaling is in agreement with previous observations in CD45-deficient mice (43, 45, 49). To assess E3/49K activity, we tested E3/49K binding can be titrated by supplementation of decoy receptors. Therefore, supernatants containing HA-49K-D64 (**Figure 4D**) and purified proteins (**Figure 4E**) were titrated and incubated with a constant amount of hCD45-Fc. Mixed reagents were incubated together with Ramos cells HA-49K-D64 binding was detected, showing that samples with decoy receptors prevented efficiently E3/49K binding at a dilution of 1:16 for the supernatant and a concentration of 4 µg per sample for the purified protein, whereas without decoy receptors E3/49K binding was still possible. These conditions were also used to characterize whether decoy receptors can restore Erk1/2 phosphorylation of HA-49K-D64 treated Ramos cells during stimulation (**Figure 4F**). HA-49K-D64 from supernatants or purifications efficiently inhibited the formation of pErk1/2 which was rescued by administration of hCD45-Fc decoy receptors.

### 3.5 E3/49K also inhibits BCR signaling in primary B cells

**Figure 5:**
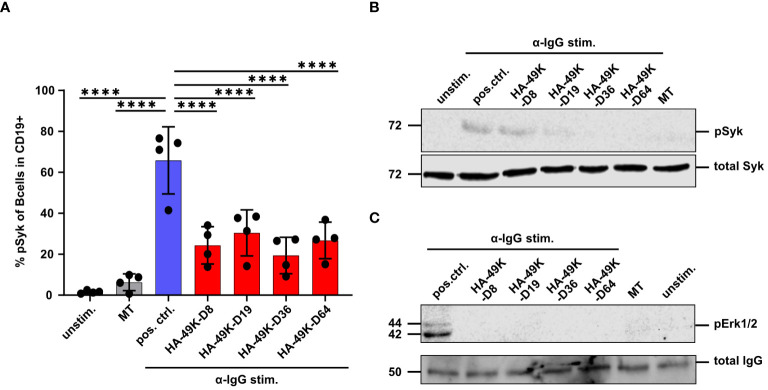
HA-49Ks also efficiently suppress the phosphorylation of signaling components during BCR stimulation in primary human B cells. Primary human B cells were isolated from PBMCs (n=4 donors, each depicted as single dots). B cells were treated with HA-49K orthologs (red) or left untreated (used as positive control, blue) and stimulated with 10 µg/ml α-IgG Abs. Unstimulated cells and 1 µg MT treated cells, as an unrelated protein control, functioned as negative controls (grey). The number of cells positive for phosphorylated Syk was measured by flow cytometry. To identify CD19+ live cells in PBMCs, cell fraction was selected and debris excluded using the forward scatter area (FSC-A) vs the sideward scatter area (SSC-A). Cell doublets that deviated from the linear correlation between the FSC-A and the forwscatter height (FSC-H) were excluded from downstream analysis. Identification of live cells was done by gating for FSC-A vs live/dead staining. B cells were identified using FSC-A vs CD19 plots. The columns indicate mean MFIs measured for B cells using the anti-phosphorylated Syk staining with error-bars depicting the standard deviation. Statistical analysis was performed using the two-way ANOVA test. Only significant differences (****P<0.0001) to the positive control are indicated in the panel **(A)**. From this setting lysates of primary B cells were generated and examined by immunoblotting for the phosphorylation of Syk **(B)** and Erk1/2 **(C)**. Total Syk and IgG served as a loading controls. One representative blot of each is shown.

Based on the impact of E3/49Ks on signaling in the human B cell line Ramos, we aimed to verify these findings using primary human B cells as targets. Antigenic stimulation induces SFK activity in B cells provoking the recruitment and activation of Syk (57, 58). Syk represents a critical factor within the BCR signaling network and functions as signal component and amplifier (59, 60). Based on these facts, we measured Syk activation by detecting pSyk using flow cytometry in α-IgG stimulated primary B cells under HA-49K treatment. The primary B cell pool was isolated from fresh blood samples and cells were treated with various HA-49K types. Following α-IgG stimulation to activate IgG positive memory B cells, pSyk was measured via flow cytometry. HA-49K treatment resulted in a clear reduction of pSyk by approximately 50% on average for all HA-49K types (**Figure 5A**). Western blot analysis from cell lysates of such treated B cells verified the previous observation of impaired Syk activity (**Figure 5B**). Comparable to **Figure 5A** residual pSyk is still detectable. Using cell lysates from processed B cells for assessing of the pErk1/2 as in the previous experiment identified an even more drastic inhibition of Erk phosphorylation by HA-49K treatment compared to the Ramos cell model (**Figure 5C**). Consistent with the above data in the Ramos cells and previous observations in CD45-deficient mice (43, 45, 49), we could demonstrate that E3/49K mediated modulation of CD45 efficiently inhibits BCR dependent Syk and MAPK signaling in primary human B cells.

### 3.6 CD45 modulation via binding to its ectodomain is unique for HAdV species D

**Figure 6:**
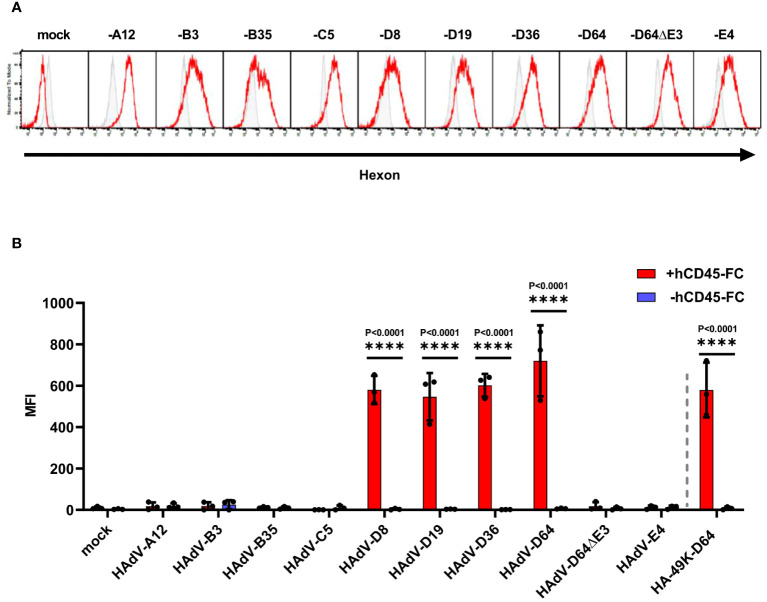
Only HAdV-D infected A549 cells bind soluble hCD45-Fc. A549 cells were infected with HAdV-A12, -B7, -B35, -C5, -D8, -D19, -D36, -D64 and -D64ΔE3 and -E4, viruses with an MOI of 5 for 24 h Efficient infection was confirmed by internal hexon protein expression (red) using 2Hx-2 mAbs in comparison to isotype control staining (grey) of infected A549 cells in flow cytometry. Displayed are representative histograms of productive infections **(A)**. Infected cells were treated with +/- (red/blue) 0.5 µg hCD45-Fc per sample. Bound CD45 molecules were detected by CD45-ECD staining using α-human pan-CD45 MEM-28 in flow cytometry. Mock infected cells as well as cells infected with HAdV-D64ΔE3 virus served as negative controls. As positive control the cell clone stably expressing HA-49K of HAdV-D64 was applied. The mean of 3 individual experiments (columns) from (dots) including standard deviation, presented as error bars, is shown. Significant differences between +/- hCD45-Fc treatment were determined using the two-way ANOVA test and are indicated in the panel **(B)**.

To date, a CD45 modulatory activity via binding to its ECD is only described for HAdV-D64 (24). As shown above, we demonstrated that E3/49Ks of further species D types tested, all shared this capability. Whether such a CD45 modulation activity can also be demonstrated for the remaining non-E3/49K expressing HAdV species remains unanswered. To address this question, we established a new methodology that allows the identification of the cell surface expression of CD45-ECD binding molecules, which can be detected by the recruitment of the soluble CD45-ECD to the cell in question. We validated this method using HA-49K producer cell clones as positive control (**Supplementary Figure 3**). This technique was applied to detect the expression of any binding activity to recombinant CD45-ECDs after productive infection of A549 cells with representatives of the different HAdV species and was controlled by staining of hexon capsomers at late times (**Figure 6A**). Apart from species D no other HAdV species exhibited such an activity (**Figure 6B**), confirming a unique immunoevasive property for HAdV of species D. Detected binding of CD45 proteins correlated with the cell surface expression patterns of E3/49K of the HAdV-D64 variants and of HA-49K of the HAdV-D64 producer cell line (**Supplementary Figure 4**). In addition, the detection of this effect could be inhibited with the pan-CD45 specific mAb GAP8.3, which competes with E3/49K for the binding site on CD45 (ref. 24 and **Supplementary Figure 5**). Taken together, our data provide evidence that the distinct CD45-E3/49K interaction is responsible for the determined binding activity specific for species D.

## 4 Discussion

In this study we extended our analysis of the HAdV-D64 E3/49K protein (22) that was shown to bind and inhibit various leukocyte functions (22, 24) to E3/49Ks of other species D types. To this end, we constructed HA-tagged versions of genetically diverse E3/49K proteins derived from HAdV-D8, -D19, -D36 and -D64. All these HA-tagged e3/49Ks bound exclusively to CD45 expressing cells (**Figure 2B**). After deletion of CD45 in T and B cell types, we did not detect significant residual binding of any of the CR1-β orthologs tested. Furthermore, the E3/49K orthologs derivedfrom different HAdV-D types tested, either associated or not with EKC, shared a similar binding activity and modulation of CD45 signaling, suggesting that it may be a common feature for probably the entire species D, since binding of E3/49K ectodomain from other types of HAdV species D has also been demonstrated (48). Moreover, we could not obtain evidence for the existence of any type-specific secondary receptor for CR1-β proteins, as previously proposed by Martinez-Martin (48), and the common binding activity, independent of the individual disease association of the corresponding virus types. This suggests that the previous assumption that certain E3/49Ks might be associated with EKC disease is unlikely (24, 48). Moreover, our results suggest that CD45 modulation via binding to its ECD is a uniquel feature of species D (**Figure 6A**). One can speculate that this may be due to a potentially superior adaptation to and better coexistance with the human population, which is thought to be the original ancestral reservoir for species D (10). In addition to being the largest and most rapidly emerging group of HAdVs, species D is the only strictly human-specific species (3, 10, 61). Since CD45-based immune evasion serves a suitable target for several pathogens (27, 62–64), we assume that our newly developed technique for detection of CD45-ECD ligands can be applied to other CD45 ligands as well, such as HCMVs UL11 (65), or utilized for the discovery of novel viral CD45 ligands.

**Figure 7:**
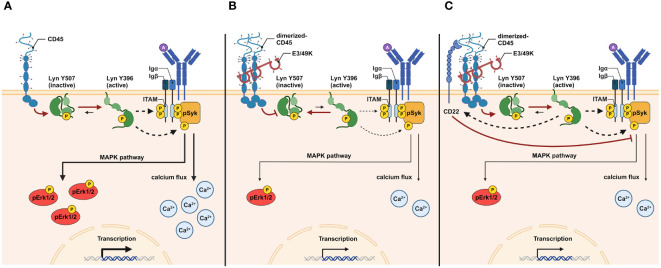
Graphical abstract of the putative functional mechanism of E3/49K action in B cells. During BCR antigen ligation, the catalytic activity of CD45 shifts the equilibrium of functional Lyn toward activated Lyn (Lyn Y396). The removal of the inhibitory phosphate group from Y507 sites primes the auto-phosphorylation of Lyn at Y396 sites to induce Lyn kinase activity. Active Lyn promotes phosphorylation of ITAMs and pITAM-attached Syk to facilitate BCR signal transduction. pSyk initiates several signaling pathways, including the MAPK pathway. pErk1/2 and the calcium flux results in transcriptional and cellular activation **(A)**. E3/49K-mediated dimerization of CD45 molecules prevents the catalytic activity of CD45. As a result, the equilibrium of functional Lyn is shifted toward inactive Lyn (Lyn Y507), increasing the activation threshold during BCR stimulation. As a result of reduced Lyn kinase activity, less pSyk, pErk1/2, and calcium flux are generated, resulting in decreased transcriptional and cellular activation **(B)**. Dimerization of CD45 by E3/49K may disrupts CD22-CD45 interaction. Active Lyn induces CD22 which enhances its inhibitory effect in reducing BCR signals by affecting pErk1/2 and calcium flux **(C)**. The figure was created with BioRender.com.

According to a recent hypothesis for the mechanism of E3/49K-mediated inhibition of CD45 by soluble E3/49K of HAdV-D64, E3/49K has two binding sites for CD45 molecules, which provokes the dimerization of CD45, causing the inhibition of CD45 function (25). The decrease of active CD45 results in increased levels of inactive Lck containing the inhibitory pY505 phosphorylation and finally an impairment of TCR signaling (25). This is in agreement with previous reports describing that during the CD45-CD45 interaction a juxta-transmembrane wedge within the CD45 molecule sterically blocks the catalytic site of its binding partner and thereby prevents SFK-substrate phosphorylation resulting in a negative regulation in T and B cell receptor signaling (66–69) (**Figure 7A**). In this regard, it can be hypothesized that B cells treated with E3/49K exhibit a phosphorylation pattern tending to inactive Lyn (**Figure 7B**), the complementary SFK in B cells, similar to inactive Lck in T cells. CD45 numbers and BCR signaling capacity are correlating with the expression of activated Lyn kinases. B cells that are CD45-deficient or have a low expression of CD45 exhibited inactive Lyn, having pronounced inhibitory phosphorylation at position Y507 and low activating phosphorylation at position Y397 (70). CD22 is an inhibitory co-receptor that contains immunoreceptor tyrosine inhibitory motifs (ITIMs) in its cytosolic segment. Phosphorylation of the ITIMs by Lyn facilitates the recruitment of the SHP-1 and SHIP1 phosphatases (71). Important for the inhibitory function of CD22 is its cell surface organization, in close proximity to the BCR it antagonizes BCR signals, sequestered from the BCR nanoclusters, BCR signaling is enhanced (72). CD45 is reported to form heteromultimeric interactions *in cis* with CD22 via the ectodomains of both proteins. Thus, this non-enzymatic function of CD45 may be involved in the cell surface regulation of CD22 independent of its catalytic domain (73). The E3/49K-CD45 interaction may disrupt the interaction between CD45 and CD22, which may enhance the inhibitory effect of CD22 (**Figure 7C**). One indication could be provided by antibody-mediated cross-linking of CD45, resulting in physical sequestration from CD22, leading to an increase in tyrosine phosphorylation of CD22 and activation of SHP-1 (74). The effect of E3/49K on CD22 function should be further analyzed in the future.

In this study we demonstrated that, despite the high structural diversity between E3/49K types (11), they share the conserved activity to bind to CD45 on all cells tested. All E3/49K types mediated inhibition of BCR- and TCR signaling, as suggested by an impairment of signaling components, which might be due to negative regulation of SFKs. Here, our primary aim was to test whether the different orthologues have comparable or divergent effects. As we found that the different orthologues from distant types of HAdV species D share most likely the binding site and act very similarly, we focused only testing basic effects for already described target cells such as T- and NK-cells. Accordingly, reduction of CD69 surface upregulation upon TCR stimulation of Jurkat cells could be reproduced for HA-49K of -D64 (24, 25) and applied to all other tested HA-49K types. Moreover, impairment of the phosphorylation of Erk1/2 in Jurkat cells could be verified (24, 25) also for the remaining HA-49Ks tested. Defective CD69 induction in T cells from CD45-deficient mice has been previously described (75). The induction of CD69 surface expression in T-cells (76, 77) has been previously linked to the MAPK pathway, providing evidence for reduced CD69 levels in combination with impaired pErk1/2 signals. However, induction of CD69 is not exclusively regulated by the MAPK pathway and can be caused from canonical NF-κB signaling as well, mediated for example by CD28 stimulation (78, 79). Thus, remaining CD69 surface expression upon HA-49K treatments could be explained independent from defects in Erk1/2 signaling (80). On the other hand, especially the above mentioned pErk1/2 signal is clearly impaired upon stimulation of T cells, as a result of treatment with HA-49Ks, replicating previous studies with E3/49K of HAdV-D64 (24, 25). Suppression of pErk1/2 acquired by negative regulation of CD45, via E3/49K, reflects interestingly the state of TCR receptor signaling events in Jurkat cell lines and T cells from mice having both defects in the expression of CD45. By this means, E3/49K treatment phenocopied the TCR signaling capacity of mice expressing only 3–7% of total CD45 (CD45low) compared to wild-type (70, 81, 82) and to some degree in CD45-deficient mice (32, 55, 56, 68).

Such phenotypic mimicry with utilization of E3/49K as tool to generate conditions under defective CD45 expression could be consequently subjected in the case of BCR signaling events and can be controlled by supplementation of E3/49K decoy receptors. Binding of E3/49K of HAdV-D64 to B cells was previously demonstrated (24), suggesting a potential immunomodulatory effect on B cells. Indeed, the HA-49K types tested here provoked a drastic impairment of pErk1/2 in Ramos and primary B cells after BCR stimulation, which is consistent with results from B cells originating from CD45-deficient (43, 45, 49) or CD45low mice (70). Comparable to data from mice lacking or having a diminished expression of CD45, we observed a dampened calcium flux response in Ramos cells during BCR stimulation upon HA-49K treatment (43, 44, 70). Accordingly, Syk activity was reduced in stimulated B cells after treatment with HA-49Ks, reflecting data from CD45-deficient mice and J558L mu m3 plasmacytoma cells (73, 83). Calcium flux events are downstream from Lyn- and Syk-derived activities. Interestingly, Syk signaling can occur, independent from SFK activity via a different pathway (83–85). Another possibility for the maintenance of pSyk and calcium flux is provided by the existence of a second distinct phosphatase called CD148, that has overlapping functions to CD45 in the initiation of BCR signaling in conventional B cells (46, 49). Clearly, to describe the detailed mode of action and functional consequences of E3/49K interaction further studies are required with expansion of detailed studies on more compelling primary B cell models.

Taken together, these data demonstrated the potential of an E3/49K mediated immunosuppression of B cells by inhibiting the BCR signaling capacity and complement the recent observations of reduced B cell activation and antibody production during E3/49K expression in porcine cells (47). Moreover, B cells were shown to be critical in the clearance of AdVs in the mouse model *in vivo* (86), our findings identify E3/49K as the first viral immunoeavasin affecting directly this important cell type. However, further investigations are required to characterize the functional effects on B cell caused by E3/49K. If an attenuated adaptive immune response is provoked as a result of E3/49K-caused effects HADV of species D could have a striking advantage compared to the other species, potentially playing a role in the dominance of species D types among HAdVs. Dampening the humoral immune response probably prevents efficient HAdV clearance, prolonging shedding of infectious viruses and thereby aid persistent infection at sites which are inefficiently controlled by cell mediated immune responses. This is consistent with the observation of higher and longer-lasting viral loads and disease progression in eye infections of species D compared to other species (87). Elaborating this further, one can suggest that treatment of E3/49K potentially causes poor proliferative response and maintenance of germinal center reactions as a result of impairment of CD45 as previously reported in CD45-deficient mice (40, 41, 45). It can be speculated that during E3/49K exposure IgM production will be mainly generated by the T cell independent B1 B cell subset, which can function independent from CD45 (88).

**Figure 8:**
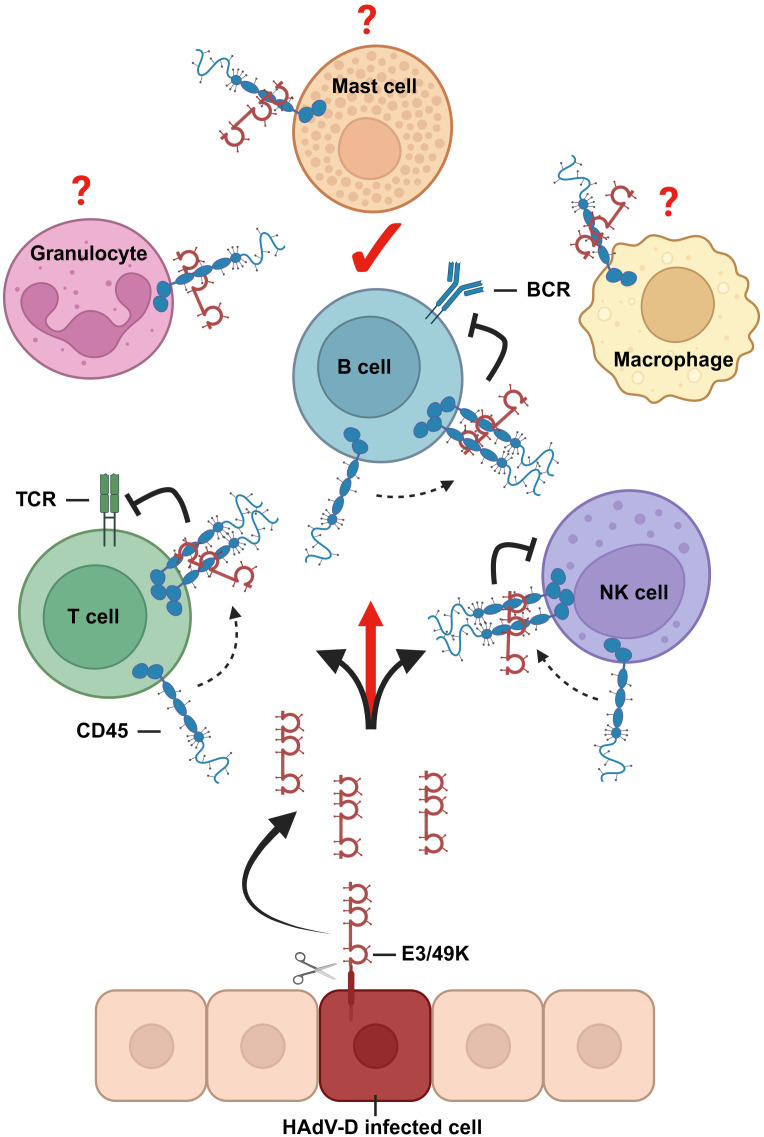
Graphical representation about E3/49K functions. CD45 modulation via binding of E3/49K proteins to its ECD is a common feature of HAdVs of species D. E3/49K ECDs are shed from infected cells and bind to and inhibit CD45 positive target cells. Based on the current hypothesis, inhibition is mediated by enforced dimerization of CD45 (25), which inhibits leukocyte receptor signaling. B cells are here identified as a new target for E3/49K-mediated immune evasion. Since there are more CD45 expressing leukocytes existing, it is hypothesized that they serve as targets for E3/49K as well. The figure was created with BioRender.com.

Based on these data, we propose that E3/49K treatment of immune cells mirrors defects in CD45 expression as in CD45-deficient or reduced expression conditions. The availability of E3/49K as tool to inhibit CD45 opens a new window to investigate CD45-specific functions which are still poorly characterized, for example in B cells or especially in leukocytes of the myeloid linage (46). This would allow further investigations directly in human cells, apart from mouse models and cells lines, to identify and characterize in greater detail direct functional impacts. Since there are more CD45 expressing cell types existing that can be bound by E3/49K (**Figure 8**), we expect that E3/49K has even more cellular targets for CD45-based immune evasion.

## Data availability statement

The raw data supporting the conclusions of this article will be made available by the authors, without undue reservation.

## Ethics statement

The studies involving humans were approved by Ethics Committee of the Albert-Ludwigs-University, Freiburg Germany (474/18). The studies were conducted in accordance with the local legislation and institutional requirements. The participants provided their written informed consent to participate in this study.

## Author contributions

AHi: Conceptualization, Data curation, Formal Analysis, Investigation, Methodology, Validation, Visualization, Writing – original draft. PC: Funding acquisition, Investigation, Methodology, Writing – review & editing. MB: Funding acquisition, Investigation, Methodology, Writing – review & editing. NK: Investigation, Methodology, Writing – review & editing. KK: Investigation, Methodology, Writing – review & editing. CN: Investigation, Writing – review & editing. MR: Resources, Writing – review & editing. AHe: Resources, Writing – review & editing. HH: Funding acquisition, Project administration, Resources, Supervision, Writing – review & editing. H-GB: Conceptualization, Resources, Supervision, Writing – review & editing. ZR: Conceptualization, Data curation, Funding acquisition, Project administration, Writing – review & editing.
