# Supplementary material for: Inhibition of B cell receptor signaling induced by the human adenovirus species D E3/49K protein
Source: Front Immunol. 2024 Jul 30;15:1432226. doi: 10.3389/fimmu.2024.1432226 (PMC11321000; doi:10.3389/fimmu.2024.1432226)
Supplement: Supplementary file 1 [file DataSheet_1.pdf]

Supplementary Material

Inhibition of the B cell receptor signaling induced by the human adenovirus species D E3/49K protein

Andreas Hildenbrand, Precious Cramer, Milena Bertolotti, Nathalie Sophia Kaiser, Kathrin Kläsener, Clara Muriel Nickel, Michael Reth, Albert Heim, Hartmut Hengel, Hans-Gerhard Burgert and Zsolt Ruzsics*

*** Correspondence:** Dr. med. Zsolt Ruzsics: zsolt.ruzsics@uniklinik-freiburg.de

A

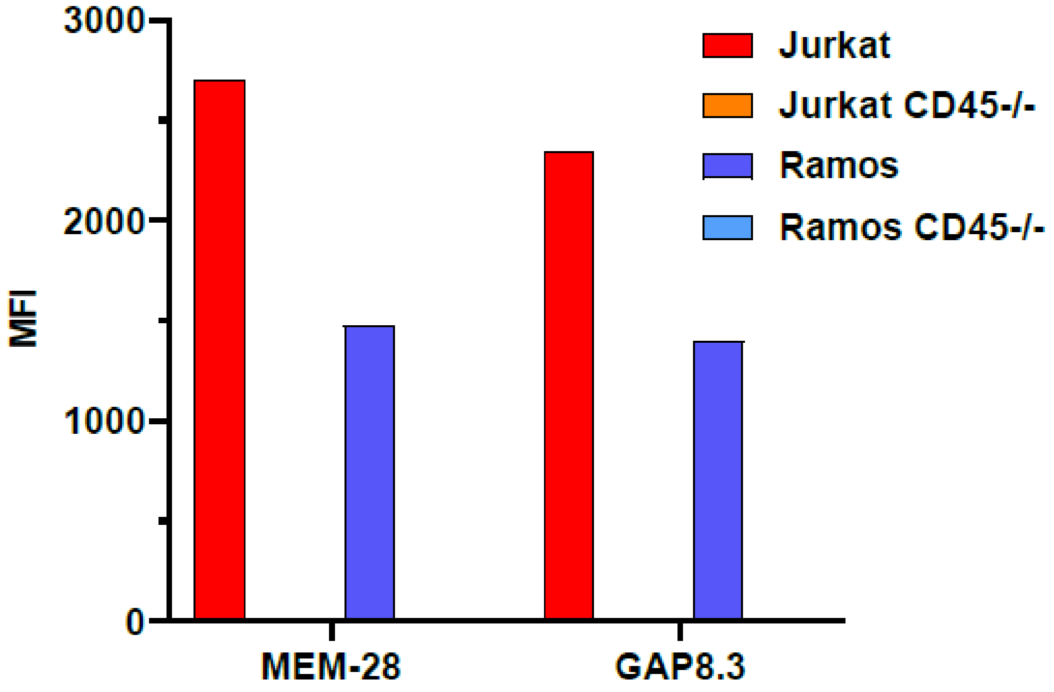
3000
2000
1000
0
MFI
Jurkat
Jurkat CD45-/-
Ramos
Ramos CD45-/-
MEM-28
GAP8.3

B

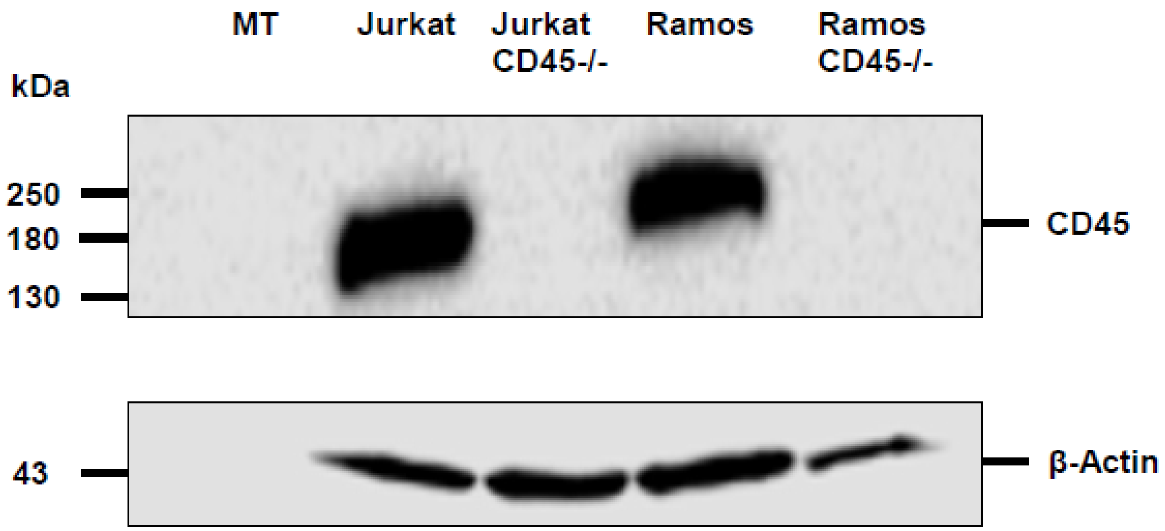
MT
Jurkat
Jurkat CD45-/-
Ramos
Ramos CD45-/-
kDa
250
180
130
CD45
43
β-Actin

Supplementary Figure 1. Identification of CD45 levels of Jurkat and Ramos cell lines
Flow cytometry measurements of CD45 cell surface expression levels for wild-type Jurkat (red) and Ramos cells (blue), and in CD45-deficient Jurkat (orange) and Ramos cells (light blue). CD45 levels were determined by staining with the two pan-specific α-human CD45 mAbs MEM-28 and GAP8.3. One representative experiment out of two is displayed (**A**). Cell lines were lysed and lysates were analyzed for CD45 expression using α-human/monkey CD45 (D9M8I) XP (Cell Signaling Technology) Ab. Detection of β-actin levels was used as loading control (**B**).

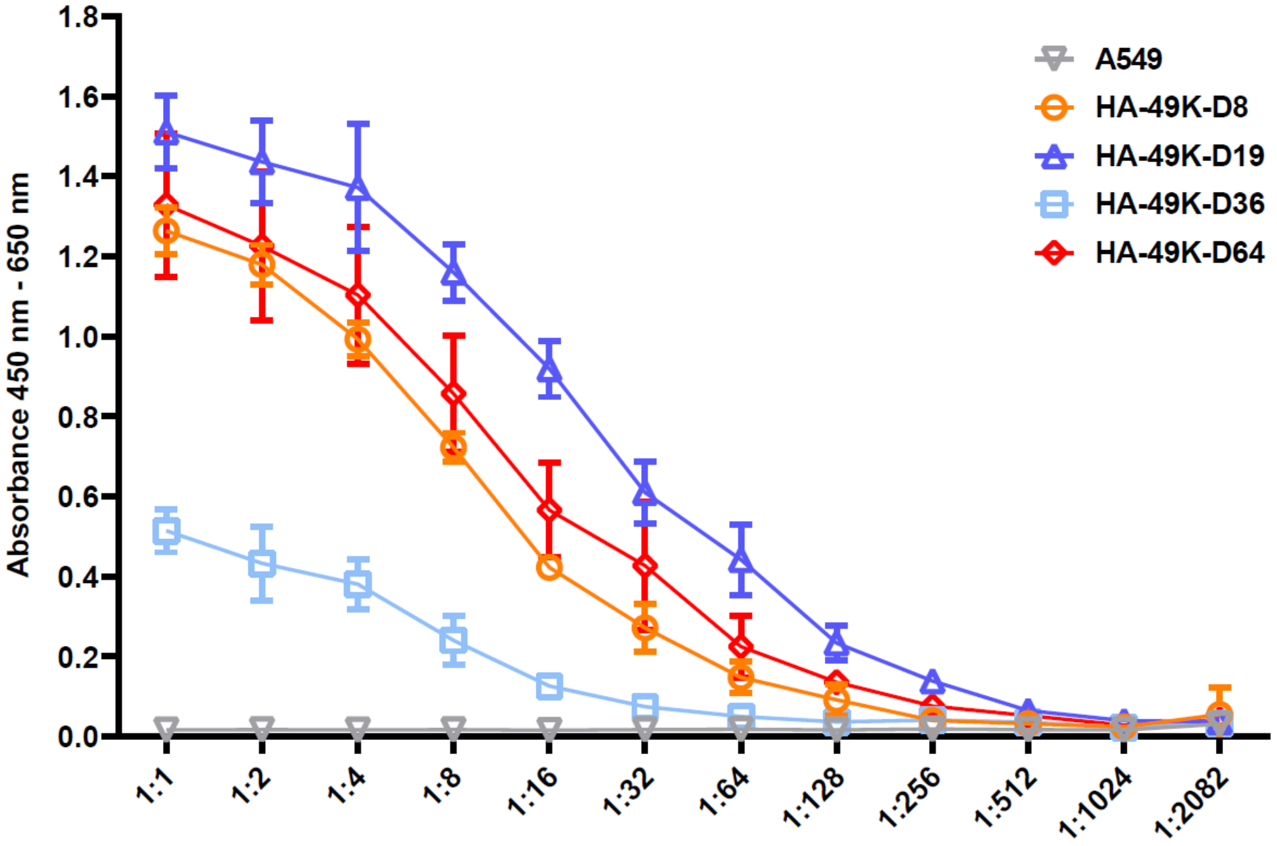

Supplementary Figure 2. Quantification of HA-49K levels in cell supernatants derived from the A549-based expression system
Cell culture supernatants from the A549-based expression system was generated as described above. HA-49K levels from supernatants of the individual producer cell clone for HA-49K of HAdV-D8 (orange), -D19 (blue), -D36 (light blue) and -D64 (red) were quantified via an α-HA based Sandwich ELISA. Supernatant from untransfected A549 were used as negative control (grey). Detection was based on its binding activity to its target receptor human CD45. 50 µl of supernatants were used per sample and diluted as indicated on the X-axis. For the ELISA Nunc MaxiSorp™ flat-bottom plates were coated with 0.25 µg/ml anti-HA Abs (both from Thermo Fisher Scientific) overnight at 4 °C, followed by blocking with 10 % goat serum (Merck AG) overnight. HA-49 supernatant dilutions were added to the plate followed by 1 µg/ml hCD45-Fc in PBS solution (50 µl per well) and incubated at least for 2 h at RT, followed by 30 min incubation with 0.16 µg/ml of a horseradish peroxidase coupled α-human IgG. Detection was performed via a colorimetric using TMB substrate (Thermos Fisher

Scientific). Reaction was stopped at 20 min with 1 M sulfuric acid. Colorimetric absorbance measurement was executed at 450 nm and a reference wavelength of 620 nm, using the ELISA reader “Infinite® M PLEX” and the “software i-control™, 2.0” from the Tecan Trading AG.

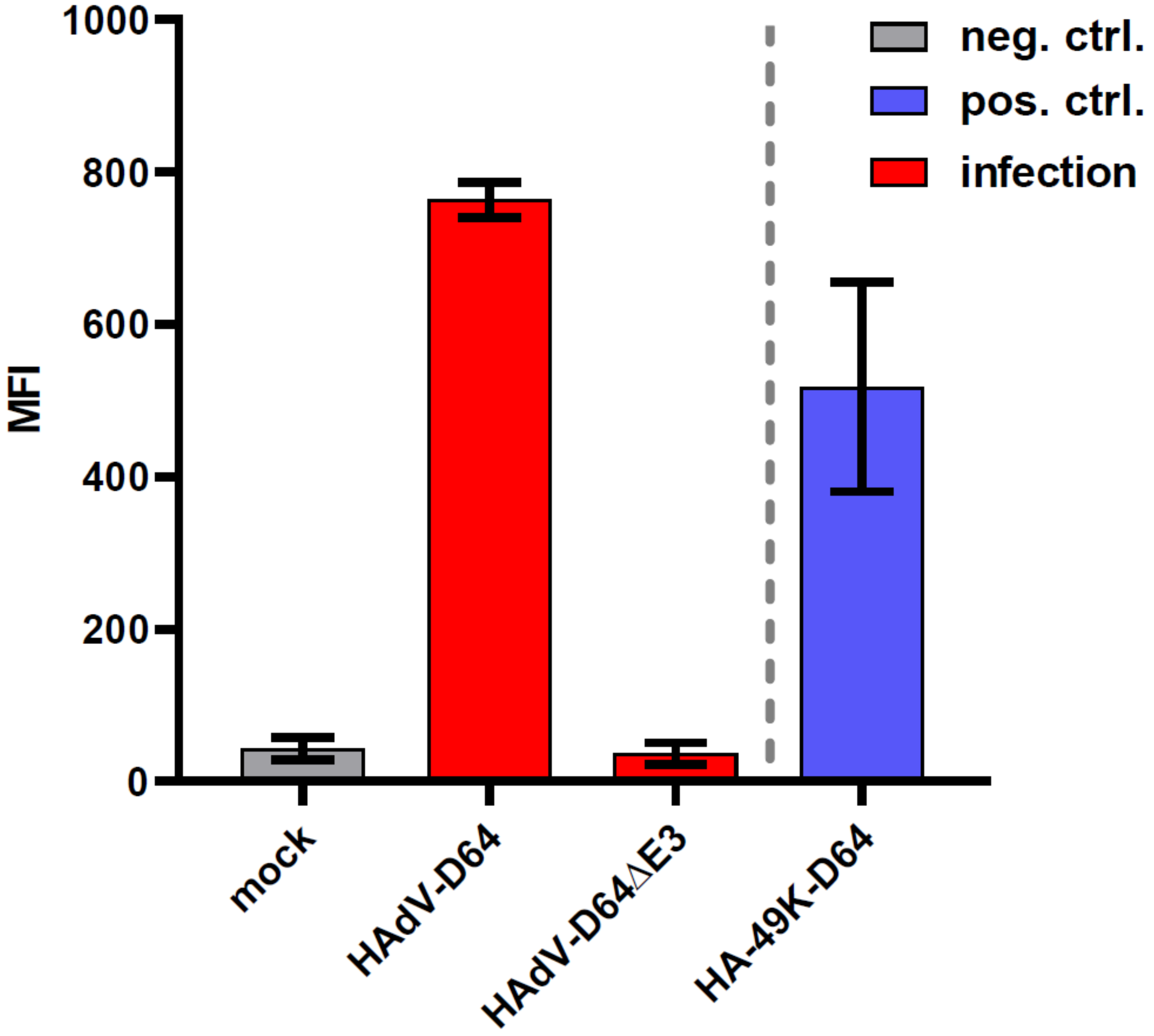

Supplementary Figure 3. Detection of cell surfaced expressed E3/49K on HAdV-D64 infected cells

A549 cells were infected with an MOI of 5 with HAdV-D64 or HAdV-D64ΔE3 (red). Cell surface resident E3/49K was detected with mAb 4D1 Ab by flow cytometric analysis. The mock infection

(grey) was performed as negative control, and a cell clone expressing stably HA-49K of HAdV-D64 was utilized as positive control (blue) in this infection assay (HA-49K of HAdV-D64 clone, separated from data derived from infection with grey dashed line). As expected, the presence of E3/49K was measured for HAdV-D64 infected cells as well as for the HA-49K stable transfected cell clone. The mean of 3 individual experiments including standard deviation is displayed.

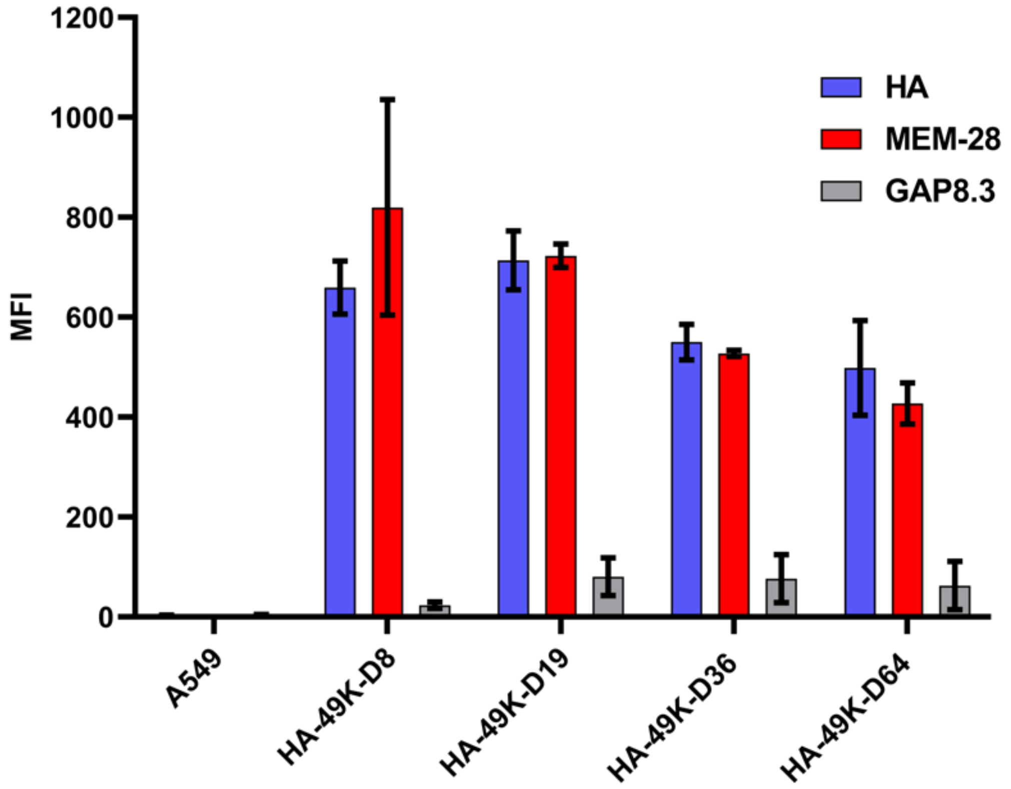

Supplementary Figure 4. Measurement of the binding activity of soluble CD45 molecules to HA-49K transfectants

Stable transfectants expressing HA-49K of HAdV-D8, -D19, -D36 and -D64, and untransfected A549 cells, were treated with 0.5 µg per sample hCD45 Fc. Bound CD45 molecules were detected with α-human pan-CD45 MEM-28 (red) and GAP8.3 (grey) Abs. For comparison, HA-49K surface expression levels of the individual cell clone was measured by α-HA staining (blue). Untransfected A549 cells, being negative in all cases, were utilized as a negative controls. Bound CD45 to the different HA-49K types with MEM-28 was possible to similar levels compared to the HA-49K surface levels. In contrast to that, only low levels of bound CD45 molecules were detectable because of the competition of HA-49Ks with the GAP8.3 Ab for a potential similar binding site on CD4524. MEM-28 allows a precise measurement of a CD45 ECD binding activity. The mean of 3 individual experiments including standard deviation, presented as error bars, is shown.

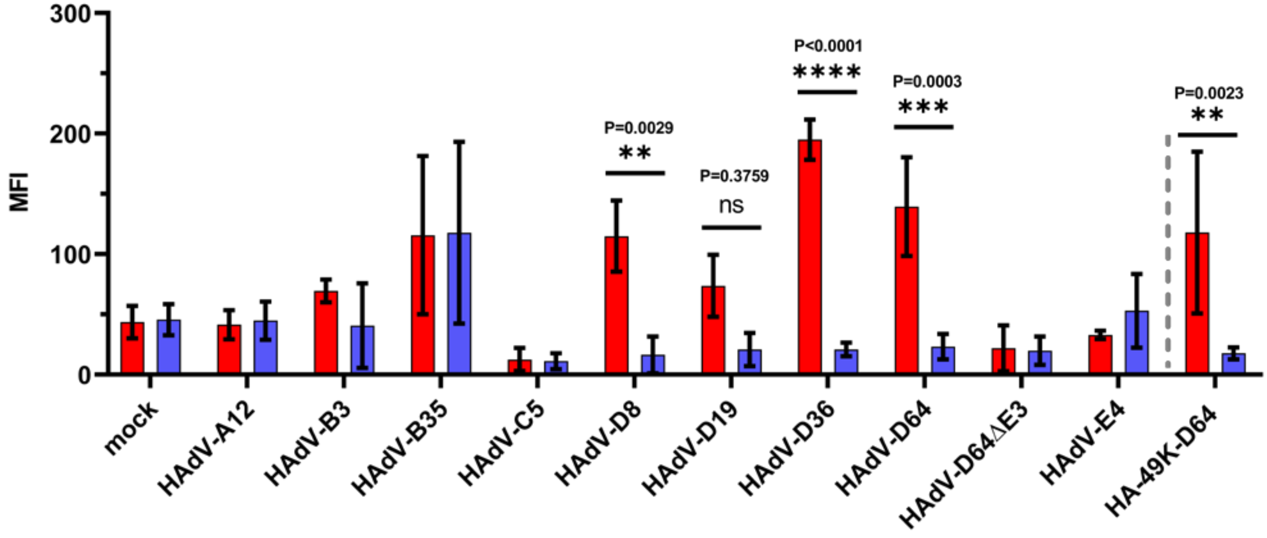

Supplementary Figure 5. CD45-E3/49K interactions are blocked with GAP8.3 anti-CD45 Abs A549 cells were infected with equal MOIs of 5 HAdV-A12, -B7, -B35, -C5, -E4, -D8, -D19, -D36, -D64 and -D64ΔE3 viruses (MOI of 5). Infected cells were treated with +/- (red/blue) 0.5 µg hCD45-Fc per sample. Flow cytometric detection of bound CD45 molecules with usage of blocking α-CD45 GAP8.3 Abs was inhibited, because of the competition between the blocking Ab and E3/49K for the CD45-binding site24. Mock infected cells as well as infection with HAdV-D64 ΔE3 virus was performed as negative controls. The cell clone expressing stably HA 49K of HAdV-D64 was applied as positive control (HA-49K -D64 clone, indicated with grey dashed line). The mean of 3 individual experiments including standard deviation, presented as error bars, is shown. Significant differences between +/- hCD45-Fc treatment were determined using two-way ANOVA test and indicated in the figure.
